# HNSCC: Tumour Antigens and Their Targeting by Immunotherapy

**DOI:** 10.3390/cells9092103

**Published:** 2020-09-15

**Authors:** Adrian von Witzleben, Chuan Wang, Simon Laban, Natalia Savelyeva, Christian H. Ottensmeier

**Affiliations:** 1 Cancer Sciences Unit, Faculty of Medicine, University of Southampton, Southampton SO16 6YD, UK; a.von-witzleben@soton.ac.uk (A.v.W.); N.Savelyeva@soton.ac.uk (N.S.); 2 Department of Otorhinolaryngology, Head & Neck Surgery, University of Ulm, 89081 Ulm, Germany; simon.laban@gmail.com; 3 Head and Neck Center, Institute of Systems, Molecular and Integrative Biology, University of Liverpool, Liverpool L69 7ZX, UK; chuan.wang@liverpool.ac.uk

**Keywords:** HNSCC, cancer antigens, cancer testis antigens, viral antigens, HPV

## Abstract

Head and neck squamous cell carcinomas (HNSCC) are a heterogeneous group of malignant tumours typically caused by alcohol and tobacco consumption, although an increasing number of HNSCC arise due to persistent infection with high-risk human papilloma virus (HPV). The treatment of HNSCC remains challenging, and the first-line setting is focused on surgery and chemoradiotherapy. A substantial proportion of HNSCC patients die from their disease, especially those with recurrent and metastatic disease. Among factors linked with good outcome, immune cell infiltration appears to have a major role. HPV-driven HNSCC are often T-cell rich, reflecting the presence of HPV antigens that are immunogenic. Tumour-associated antigens that are shared between patients or that are unique to an individual person may also induce varying degrees of immune response; studying these is important for the understanding of the interaction between the host immune system and the cancer. The resulting knowledge is critical for the design of better immunotherapies. Key questions are: Which antigens lead to an adaptive immune response in the tumour? Which of these are exploitable for immunotherapy? Here, we review the current thinking regarding tumour antigens in HNSCC and what has been learned from early phase clinical trials.

## 1. Introduction

More than 2.5 million people worldwide are affected by head and neck squamous cell carcinoma (HNSCC), and more than 379,000 deaths per year can be attributed to this disease [1,2]. HNSCC is a heterogeneous group of cancers arising from the mucous membrane of the oral cavity, pharynx, and larynx, which is mainly caused by tobacco and alcohol consumption [3]. In Southern and South-Central Asia, HNSCC and in particular oral squamous cell carcinoma (OSCC) are linked to smokeless tobacco and paan, causing both OSCC and oral premalignancy [4]. Alongside tobacco, areca nut included in betel quid is also a known carcinogen, and the mixture of tobacco, areca nut, and slaked lime forms a potent carcinogenic combination. However, there is a biologically distinct subgroup of HNSCC that is caused by the high-risk human papillomavirus type 16 (HPV16), with an incidence of 25.9% worldwide irrespective of other carcinogens [5,6,7,8]. These tumours are typically localised in the oropharynx (OPSCC) and ≈50% tonsillar carcinomas are driven by HPV [5]. Despite commonly presenting with locally advanced disease and involvement of regional lymph nodes, these patients have a five-year overall survival (OS) rate of 70–80%. In contrast, patients with HPV16^neg^ cancer have a worse five-year survival at ≈40–60% [9,10,11,12,13,14,15]. Primary treatment options of HNSCC are surgery, radiotherapy and chemotherapy, often in combination. Despite their widespread use, such treatment is frequently ineffective, but nonetheless can substantially reduce the patient’s quality of life. In particular, salvage surgery often has to be extensive and is mutilating for the patient. A better understanding of the underlying biology of HNSCC in recent decades has led to studies testing new, more targeted treatment options with less side effects and less consequential damage than the standard therapy.

The improved survival of HPV^pos^ OPSCC and the observation that antiviral T cells are common in these tumours suggest that adaptive immune responses play an important protective role in HNSCC. As early as 1863, the interaction of lymphoid cells and neoplastic cells was described by Rudolf Virchow [16]. In the last decades, these interactions were investigated further, and the critical influence of lymphocyte infiltration on patient survival was confirmed in many cancer types [17,18]. It is now recognised that a deregulated immune system contributes to carcinogenesis and tumour growth, which is epitomised in the reformulation of the ‘hallmarks of cancer’ by Hanahan et al. [19]. The human immune system can contribute inflammation-stimulatory and inflammation-inhibitory factors. In health, there is a balance between these factors, and acute inflammation is only triggered in case of injury or infection. However, in cancer, this balance is shifted towards immune suppression [19]. In order for the immune system to become activated and for an adaptive immune response to develop and mature, T cells must recognise that something is amiss. One important hurdle is that the features that set cancer cells apart from healthy cells are nonetheless derived mostly from subtle changes in the quality or quantity of molecules found in the normal cells. Molecules that are shared with normal cells ‘self-antigens’ and are altered only in quantity are less likely to be visible to immune attack, as our immune system is geared towards ‘self-tolerance’. Molecules that are more accessible to immune attack are those that derive from mutated or truly foreign molecules not previously present in the host. This, the additional immune evasion that malignant disease causes over time and the inhibition of inflammation in the cancer microenvironment, constitute hurdles for successful immunotherapy [20,21].

Among the features that predict for good survival and/or successful (immuno-) therapy is the presence of tumour immune infiltrates that recognise cancer-associated antigens [22,23,24], providing histological evidence that the patient’s immune system is attempting immune attack. The impact on patient survival was found to be independent of the treatment regimens [25,26]. The presence of an adaptive immune response can be assessed by determining the number of tumour-infiltrating lymphocytes (TILs) in a tumour, in particular the number of cytotoxic CD8^+^ T cells. Published data support that patients with TIL^high^ tumours have better survival. In ≈80–85% of cases, HPV^pos^ HNSCC are infiltrated by high numbers of TILs, and these patients have the best survival rates [24,27]. In contrast, the minority of TIL^low^ HPV^pos^ OPSCC have a disease-related survival similar to that of HPV^neg^ OPSCC [24]. While it is thought that HPV-reactive T cells dominate and are protective, tumour-infiltrating lymphocytes and circulating T cells targeting non-viral HNSCC antigens have been observed [28]. These data predict that stimulating immune responses against immunogenic antigens will be useful for the patient and enable us to further identify propter hoc for which patients we might be able to boost immune responses and shift the balance towards immune protection.

The increasing understanding of tumour immunology has led to intense preclinical and clinical research to develop and to test new immuno-therapeutics [21,29,30]. The lowest bar is to activate a T-cell response that is already established but is not yet sufficient to control the cancer clinically. Once T cells are activated through their T-cell receptor (TCR), they upregulate inhibitory molecules, which in turn limit the effector function in a negative feedback loop. The name that has been coined for such molecules that control and regulate adaptive immune responses is “checkpoint molecules”. They can in many instances be targeted by antibodies, and the clinically most effective strategy that has emerged to date is the use of anti-PD-1 (programmed cell death protein 1) and anti-PD-L1 (programmed death ligand 1) antibodies [31]. By binding to PD-1 on the T cell or PD-L1 on the partner cell, such antibodies block the PD-1/PD-L1 interaction, release T-cell inhibition, and thus have the potential to allow immunological cancer control. Two anti-PD-1 antibodies, pembrolizumab and nivolumab, are approved for recurrent and metastatic HNSCC [32,33]. While these drugs can have a dramatic and durable benefit for individual patients, treatment is useful only for a minority of < 20% after the failure of standard chemotherapy [29,34]. Therefore, it is intuitive to think that the lack of naturally occurring anti-cancer immune responses will play an important role in this failure, which is reflected morphologically in a low number of TIL. In HPV^neg^ HNSCC, 85% of patients have such TIL^low^ cancers [9,24,27], suggesting that an induction of T cells that are tumour-reactive and can home to the cancer tissue is a key hurdle for improving cancer immunotherapy. This in turn argues that educating the immune system by vaccination or by the transfer of antigen-specific cells will be a critical step for most patients with recurrent HNSCC.

For the following review, we searched PubMed, clinicaltrials.gov, and the Japanese UMIN (University hospital Medical Information Network) clinical trials registry for HNSCC-related publications and trials that examined antigen-specific immune targeting of HNSCC. We included other studies of these antigens where relevant for the understanding of context.

## 2. Current Status of Antigen-Specific Immunotherapy in HNSCC

**Figure 1:**
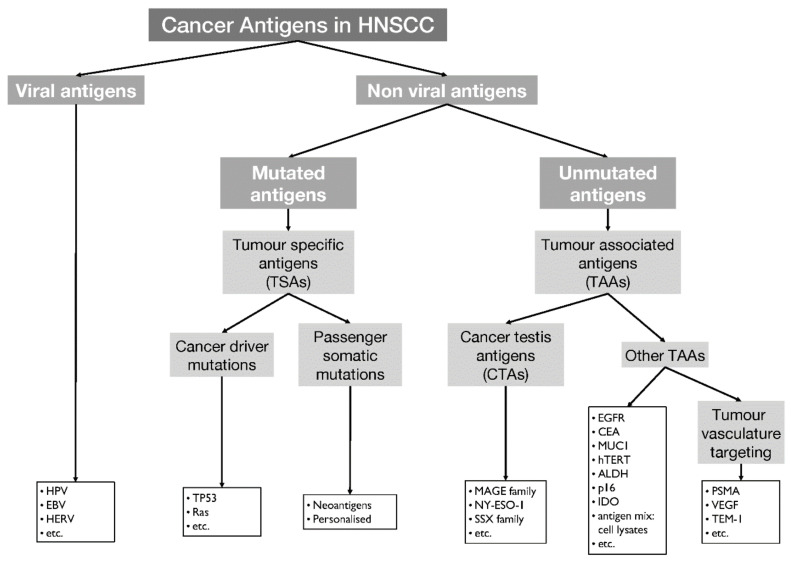
Overview of cancer antigens that are potential therapeutic targets in head and neck squamous cell carcinoma (HNSCC). TSA = tumour-specific antigen, TAA = tumour-associated antigen, CTA = cancer testis antigen, HPV = human papilloma virus, EBV = Epstein–Barr virus, HERV = human endogenous retroviruses, MAGE = melanoma-associated antigen, NY-ESO-1 = New York esophageal squamous cell carcinoma-1, SSX = synovial sarcoma X, EGFR = epithelial growth factor receptor, CEA = carcinoembryonic antigen, MUC1 = mucin-1, hTERT = human telomerase reverse transcriptase, ALDH = aldehyde dehydrogenase, IDO = indolamin-2,3-dioxygenase, PSMA = prostate-specific membrane antigen, VEGF = vascular endothelial growth factor, TEM-1 = tumour endothelial marker 1.

A key to the selective targeting of cancer cells while sparing healthy cells in the patient is the ‘training’ of T cells to recognise differences between cancer cells and their normal cellular counterparts. Specific immune recognition is encapsulated in the term ‘antigen’, and cancer antigens then are those molecules that enable the immune system to distinguish between ‘healthy’ and ‘cancer’. Cancer antigens can be classified into viral antigens, antigens derived from new, mutated genomic sequences—neoantigens—and targets that derive from unmutated proteins and that are either unique or specific for tumours (tumour-specific antigens, TSA) or that are differentially expressed in tumour cells and less so in normal cells—tumour-associated antigens (TAAs) [35]. In some instances, TAAs are related to the particular feature of a cell type and are retained in the malignant counterparts; examples are molecules related to the production of pigment, such as tyrosinase in melanocytes and melanomas (differentiation antigens) or prostate-specific antigen (PSA) in prostate and prostate cancer [36]. For molecules that are expressed during embryogenesis and lost in healthy adult cells but re-expressed after malignant transformation, the term ‘carcinoembryonic antigen’ is used. Antigens that are expressed only in the testes and in cancer cells form a separate group of TAAs: cancer testis antigens (CTAs). TAAs are found in both healthy and malignant cells as the result of a differential (higher) expression of a protein in cancer cells compared to healthy cells. In contrast, TSA are only expressed in tumour cells. Excellent examples of these are antigens, which are derived from the mutational activity that leads to transformation and can lead to the expression of tumour neoantigens. For these to be accessible for T-cell attack, the mutation has to be (1) transcribed, (2) translated, and (3) processed into peptides, which then are loaded onto major histocompatibility complex (MHC) class I or II proteins for presentation [37,38]. Tumour neoantigens are derived from the minority of mutations that pass those hurdles. Intriguing targets that may be exploitable for immunotherapy and specifically vaccination are antigens that are expressed on newly formed blood vessels; even though these are not malignant themselves, they can express targets for immunotherapy such as glutamate carboxypeptidase II (GCPII, also known as prostate-specific membrane antigen, PSMA) [39,40]. Figure 1 shows an overview of HNSCC-associated antigens.

## 3. Virus-Derived Tumour Antigens

### 3.1. Human Papilloma Virus, HPV

Human papilloma viruses are a group of DNA viruses that commonly cause infections in humans; certain types can cause neoplasia. In 1976, zur Hausen proposed that these viruses could cause cervical cancer and identified HPV16 and HPV18 in cervical cancer eight years later [41,42]. He and his team were also the first to demonstrate the existence of HPV in human tongue carcinomas [43].

HPVs can infect squamous epithelial cells through minor abrasion and direct contact at the cervix and the mucous membrane of the oropharyngeal region. HPV^pos^ OPSCC specifically is largely driven by HPV type 16 and is only rarely caused by other high-risk HPV types. This is in contrast to cervical cancer where HPV18 is a common causative agent. Most HPV infections are transient, but if the virus remains in a latent form, this can trigger transformation [44,45]. The important role of HPV16 in the pathogenesis of OPSCC has been recognised in the last decade [46,47,48,49,50]. We now understand that HPV16 can cause HNSCC in patients in the absences of the classical risk factors tobacco and alcohol, and HPV-driven HNSCC has a high frequency in developed countries [6,51,52,53,54,55].

The key event connecting HPV and carcinogenesis is the integration of viral DNA into the human genome. HPV16-coding regions consist of eight open-reading frames: E1, E2, E4, E5, E6, E7, L1 and L2. The antigens E6 and E7 are oncoproteins that deactivate the tumour suppressors p53 and pRb respectively, leading to a loss of cell cycle control [56]. E2 protein regulates E6/E7 expression by controlling their transcription. E5 has been shown to have an anti-apoptotic role; it contributes to the early stages of oncogenesis by cooperating with E6 and E7 to immortalise cells. E5 can downregulate MHC expression and enable infected cells to escape from immune recognition [57]. As a result of the functional inactivation of pRb by HPV E7 protein, more p16 (*CDKN2A*) is expressed. The normal function of p16 is the negative regulation of the cell cycle and inhibition of cyclin-dependent kinases 4 and 6 (CDK4, CDK6). When pRb is inactivated, p16 accumulates but cannot block the cell cycle. Immunohistochemical staining for p16 is used in the clinical diagnosis of OPSCC, but ≈20% of p16 overexpressing cancers are not driven by HPV, and additionally, a subset of HPV-driven cancers do not upregulate p16 [58].

Specific T-cell responses against HPV16 are found in HPV16^pos^ HNSCC patients and CD8^+^ T-cell responses, especially for E6 [59]. The density of TILs is easily detectable by histological assessment, and the correlation with survival was already noted in the introduction. A published study showed that the viral antigens trigger T cell responses, leading also to improved clinical response to standard therapy [60]. It is now thought that the recruitment of HPV16-specific T cells can enhance the effect of standard oncological treatments, offering a functional link to better prognosis of HPV-driven HNSCC [24]. Markers of T cell function are important: tumour-infiltrating T cells that have upregulated PD1 were more often found in HPV16^pos^ tumours accompanied by better survival [24,61], and this is understandable, given that PD1 expression is the consequence of the engagement of a T cell with its (cancer) target. A pooled analysis of HNSCC trials showed that patients with HPV^pos^ HNSCC benefit more from a PD-1/PD-L1 treatment than those with HPV^neg^ disease [62].

The immunogenicity of HPV antigens is being exploited in prophylactic vaccines: they induce antibodies that capture extracellular virus and prevent infection: Cervarix™ (a bivalent HPV16/18 vaccine, GlaxoSmithKline), Gardasil™ (a quadrivalent HPV 6/11/16/18 vaccine), and more recently Gardasil^®^9, containing HPV6, 11, 16, 18, 31, 33, 45, 52, and 58 (Merck Sharp and Dohme). These vaccines are beneficial in preventing infection; they target the late HPV antigen L1 by forming virus-like particles, which induce neutralising antibodies in the vaccinated patient. However, in HPV^pos^ HNSCC, the HPV antigens are expressed intracellularly and therefore are not readily accessible to antibodies. T cell-inducing vaccines are therefore needed. Traditionally, the main ‘cancer cell killers’ have been thought to be CD8^+^ T cells, and the focus in vaccinology has been to stimulate such cells. More recently, both in infectious disease and cancer, cytotoxic CD4^+^ T cells have been described, which can remove cells in an MHC II restricted fashion [63]. Therefore, it seems likely that the induction of both CD8^+^ and CD4^+^ T cells will be useful also in the control of HPV^pos^ HNSCC, which express both MHC I and MHC II [64].

As viral antigens are ‘foreign’ to the human immune system, no central tolerance is expected, making these viral antigens attractive targets for vaccination. HPV E6 and E7 have been considered to be the main cancer-driving oncogenes; therefore, they have been targeted by different vaccination approaches.

At least four different peptide vaccines are in development in clinical trials targeting HPV E6 and/or E7 (Table 1). One of these, the ISA 101 vaccine trial, has already reported outcomes. Using long, rather than minimal MHC I or MHC II restricted peptides together with a proprietary adjuvant, this vaccine is thought to induce both CD4^+^ and CD8^+^ T cells. The investigators showed that a combination of anti-PD-1 treatment with vaccination led to a promising response rate of 33% in oropharyngeal cancer (NCT02426892, Table 1) [65]. Another Phase 2 study is active for HNSCC, but currently not recruiting (NCT03258008, Table 1). GL-0817 (biropepimut-S) and GL-0810 are peptide vaccines against melanoma antigen-A3 (MAGEA3) and HPV16 E7, respectively. Vaccination was well tolerated and demonstrated immunogenicity in the majority of patients in a Phase 1 study [66] (no National Clinical Trial (NCT) number available). Another study investigated a MAGEA3 and HPV16 trojan vaccine in HNSCC and could detect antigen-specific TILs and peripheral T cells with no effect on the clinical outcome of the patients (NCT00257738, Table 1, see also CTAs section) [67]. However, until now, no peptide vaccine has been approved for clinical use.

A modified vaccinia Ankara (MVA)-based vaccine (TG4001, tipapkinogene sovacivec) and a bacterial vector encoding HPV E6/7 antigens (axalimogene filolisbac [AXAL] or ADXS11-001) are in clinical trials. In patients with cervical intraepithelial neoplasia (CIN), TG4001 led to promising results of 36% partial response or the complete resolution of CIN2/3 [68]. An ongoing study is investigating the effect of TG4001 with or without avelumab (anti-PD-L1 antibody) in HNSCC (NCT03260023, Table 1). The primary data, presented at the ESMO (European Society for Medical Oncology) meeting in 2019, show that three out of six patients demonstrated durable clinical responses, and the combination therapy led to a shift from an immune cold to an immune hot tumour microenvironment [69]. The bacterial vector ADXS11 is a listeria monocytogenes immunotherapy targeting HPV16 E7. This was investigated on its own or in combination with cisplatin in cervical cancer patients. The study showed similar median progression-free survival and similar overall response rates in both groups [70]. So far, the datasets in HNSCC patients are too small to assess immunogenicity or efficiency (NCT02002182, NCT02291055, Table 1). Other current trials without data so far testing peptide vaccines or HPV peptide pulsed peripheral blood mononuclear cells (PBMCs) are included in Table 1 (NCT02821494, NCT00019110, NCT02865135).

A DNA vaccine (MEDI-0457, previously INO-3112)) is currently in evaluation in Phase 1/2 and Phase 2 studies. The vaccines target HPV16 and HPV18 E6 and E7 antigens and are in evaluation in patients with HNSCC (NCT04001413, NCT03162224, Table 1). The first prospective clinical study using MEDI-0457 in HPV^pos^ HNSCC showed a durable HPV antigen-specific peripheral and tumour immune response (NCT02163057, Table 1) [71].

In our center, an ongoing HPV vaccine trial targeting HPV16 E6 and E7 (NCT03418480, Table 1) is investigating an RNA vaccine delivered intravenously. This vaccine appears to be safe in HNSCC patients, but no immunological or clinical data are available yet. The RNA vaccine platform has been reported to generate substantial CD4^+^ and CD8^+^ T cell immune response that appear to be linked to clinical responses [72].

In addition to HPV16 E6 and E7 as vaccination target, HPV16 E2 and E5 are other potential target antigens for HPV-associated cancers. E2 has already been successfully targeted using an MVA E2 recombinant vaccinia virus in anogenital intraepithelial lesions with complete elimination in 89.3% of female (total of *n* = 1176) and 100% of male (total of *n* = 180) patients [73]. A number of additional vaccines targeting E5 are in preclinical development [74,75,76,77]. However, no clinical data on targeting E2 and E5 in head and neck cancer are available.

Another approach is to use the patient’s own T cells as treatment. These are harvested from the cancer tissue by surgical procurement or from the blood if sufficient numbers of circulating antigen-specific cells exist. Then, the cells can be expanded or modified ex vivo and infused back into the patient as treatment. Expanded cells can also be modified before re-infusion, for example by transfecting a high-affinity tumour antigen-specific TCR [78]. In contrast, in chimeric antigen receptor (CAR) T-cell therapy, the intracellular and transmembrane domains of the artificial CAR are fused to an antibody fragment that binds a specific antigen on the cell surface. These CAR-T cells lead to a new type of cell, which behaves functionally similar to a T cell (cytokine release and cytotoxicity) but recognises target cells such as a B cell or antibody [78]. Such approaches have been revolutionary in B cell malignancies [79] but are in their infancy in solid cancer. One Phase 2 study is currently investigating TCR T-cell therapy in HNSCC patients by targeting HPV16 E6 (NCT03578406, Table 1) and two other Phase 2 studies by targeting HPV E7 (NCT04044950, NCT04015336, Table 1)

Since two anti-PD-1 targeting checkpoint inhibitors (nivolumab, pembrolizumab) have been approved, an increasing number of clinical trials are evaluating combinations of HPV16 vaccines with anti-PD-1 antibodies (e.g., NCT03618953, NCT04180215, NCT04001413, NCT03162224, Table 1). Early data from non-HNSCC vaccines suggest that an increased clinical response rate may be expected if vaccines are combined with anti-PD-1 antibodies [80,81].

**Table 1:** Overview of completed and ongoing clinical trials targeting viral antigens in HNSCC patients.

Drug/Study	Target	Type	Antigen	Additional Drug	Study Phase	Study Start	Patients Estimated/Recruited	HNSCC Patients Enrolled	Study Identifier	Status	Immune Response	Clinical Responses	Ref.
MEDI-0457 (INO-3112)	HPV16/18 E6/E7	DNA	Viral Ag	durvalumab	Phase 2	June–19	66/na	all	NCT04001413	Recruiting			
				single treatment	Phase 1/2	June–14	25/22	all	NCT02163057	Completed	Specific peripheral/tumour immune response		[71]
				durvalumab	Phase 1/2	June–17	50/35	all	NCT03162224	Active, not recruiting			
HARE-40	HPV16 E6/E7	RNA	Viral Ag	single treatment	Phase 1/2	April–17	44/na	na	NCT03418480	Recruiting as of 09/2020			
DPX-E7	HPV16 E7	peptide	Viral Ag	single treatment	Phase 1/2	December–16	44/11	na	NCT02865135	Active, not recruiting			
ISA101/101b	HPV16 E6/E7	peptide	Viral Ag	nivolumab	Phase 2	December–15	28/34	*n* = 22	NCT02426892	Active, not recruiting	Induce CD4/CD8 T cells	33% response	[65]
				utomilumab	Phase 2	April–18	44/27	all	NCT03258008	Active, not recruiting			
ISA201 (Hespecta)	HPV16 E6/E7	peptide	Viral Ag	single treatment	Phase 1	March–15	24/na	na	NCT02821494	Unknown			
ADXS11-001 (ADXS-HPV)	HPV16 E6/E7	bacterial vector	Viral Ag	single treatment	Phase 2	December–13	30/15	all	NCT02002182	Active, not recruiting			
				durvalumab	Phase 1/2	April–15	66/na	na	NCT02291055	Active, not recruiting			
MG1-E6E7, Ad-E6E7	HPV E6/E7	viral vector	Viral Ag	atezolizumab	Phase 1	June–18	75/na	na	NCT03618953	Active, not recruiting			
TheraT^®^ Vector(s)HB-201/HB-202	HPV16 E6/E7	viral vector	Viral Ag	nivolumab	Phase 1/2	December–19	140/na	na	NCT04180215	Recruiting			
TG4001	HPV16 E6/E7	MVA	Viral Ag	avelumab	Phase 1/2	September–17	52/na	all	NCT03260023	Recruiting	TME change from immune cold to hot	50% response	[69]
HPV E6/E/peptides pulsed PBMC	HPV16 E6/E7	peptide pulsed PBMCs	Viral Ag	single treatment	Phase 1	November–95	na/na	na	NCT00019110	Completed			
HPV E7-specific TCR T cells	HPV16 E7	TCR T cell	Viral Ag	single treatment	Phase 2	August–20	180/na	all	NCT04044950	Recruiting			
HPV E7-specific TCR T cells	HPV16 E7	TCR T cell	Viral Ag	single treatment	Phase 2	July–20	180/na	all	NCT04015336	Recruiting			
HPV E6-specific TCR-T cells	HPV16 E6	TCR T cell	Viral Ag	single treatment	Phase 1	September–18	20/9	na	NCT03578406	Recruiting			
GL-0810 (HPV16) and GL-0817 (MAGE-A3)	MAGEA3 and HPV16	peptide	Viral Ag/TAA (CTA)	single treatment	Phase 1	Na	na/16	*n* = 16	na	Completed	T cell and antibody responses observed	Well tolerated	[66]
Trojan	MAGEA3 and HPV16 E7	peptide	Viral Ag/TAA (CTA)	single treatment	Phase 1	November–05	90/5	*n* = 5	NCT00257738	Completed	Induction of viral/CTA-specific T cells	Acceptable toxicity	[67]
EBV-LMP-2	EBV	peptide	Viral Ag	single treatment	Phase 1	February–04	na/99	na	NCT00078494	Completed	Higher proportions of CD3 + CD4^+^ T cells	Well tolerated	[82]
MVA Vaccine encoding EBV proteins	EBV	MVA	Viral Ag	single treatment	Phase 1	March–05	22/16	*n* = 16	NCT01147991	Completed	Increased circulating CD4 T cells, and antigen-specific T cells		[83]
MVA EBNA1/LMP2	EBV	MVA	Viral Ag	single treatment	Phase 2	March–10	37/25	all	NCT01094405	Active, not recruiting			
Autologous EBV specific Cytotoxic T cells	EBV	T cells	Viral Ag	gemcitabine, carboplatin	Phase 3	July–14	330/na	all	NCT02578641	Active, not recruiting			
Tabele-cleucel	EBV	T cells	Viral Ag	pembrolizumab	Phase 1/2	November–18	60/na	all	NCT03769467	Recruiting			
EBV-specific adoptive T cells	EBV	T cells	Viral Ag	single treatment	Phase 1	February–07	28/28	all	NCT00431210	Completed	Not specified	Only 1/28 patients had complete response	[84]
				single treatment	Phase 2	January–09	20/na	all	NCT00834093	Active, not recruiting			
EBV-TCR-T cells (YT-E001).	EBV	TCR T cell	Viral Ag	single treatment	Phase 2	October–18	20/na	all	NCT03648697	Recruiting			
EBV specific-TCR-T cells	EBV	TCR T cell	Viral Ag	single treatment	Phase 1	August–19	27/na	all	NCT03925896	Recruiting			
LMBP2-specific TCR-T cell	EBV	TCR T cell	Viral Ag	single treatment	Phase 1/2	September–20	20/na	all	NCT04509726	Not yet recruiting			
CD137L-DC-EBV-VAX	EBV	Dendritic cells	Viral Ag	single treatment	Phase 1	August–17	55/na	all	NCT03282617	Recruiting			

### 3.2. Epstein–Barr Virus, EBV

Epstein–Barr virus (EBV) is a double-stranded DNA virus that leads to persistent infection with episodic reactivation. EBV can transform cells after infection through the expression of different virus-specific genes [85]. The oncogenic potential is not only limited to B cells, in which it can lead to lymphoproliferative disorders, particularly in immunosuppressed individuals, and to high-grade lymphomas. EBV can also transform epithelial cells and cause nasopharyngeal carcinoma. EBV was first identified in 1964 in a Burkitt lymphoma cell line by Epstein et al. [86]; the association of EBV with nasopharyngeal cancer (NPC) was identified soon after in 1966 [87]. The incidence of NPC is variably distributed across the world [88] with low incidence rates in the Western world and higher levels in South-Eastern Asia; the highest incidence is observed in Southern China. Almost all undifferentiated nasopharyngeal carcinomas are EBV-associated, and the viral genome can be found in every cancer cell [89]. During latency I/II, EBV gene expression is thought to be causal for transformation. The expression of Epstein–Barr virus (EBV) nuclear antigen 1 (EBNA1), non-coding Epstein-Barr virus (EBV)-encoded RNAs (EBERs) and microRNAs from BamHI A rightward transcripts (BART-miRs) as well as latent membrane protein 2 (LMP2) expression is typical [90,91]. LMP1 specifically acts by activating the NF-κB signalling pathway, which is a characteristic of NPC [92].

EBV-specific immunotherapies including T-cell immunotherapies have been and are being tested in patients with NPC. Clinical results are encouraging with sustained responses reported [93], but no cellular therapeutics have been approved to date. Virus-specific T cells grown ex vivo and re-infused into the patients are under investigation in a Phase 3 trial sponsored by Tessa Therapeutics (NCT02578641, Table 1), from which results are awaited. Here, after randomisation, peripheral blood from the patient is collected and used to generate a patient-specific cytotoxic T-cell (CTL) line. These T cells are transferred back to the patient with the aim of controlling EBV-infected tumour cells, following systemic chemotherapy with gemcitabine and carboplatin. The control patient cohort receives standard cytotoxic chemotherapy. The study builds on a Phase 2 study investigating EBV-specific CTLs (EBV-CTLs) in combination with chemotherapy (gemcitabine and carboplatin), which showed a response rate of 71.4% with three complete responses and 22 partial responses in a total of 35 patients receiving the treatment [94]. A separate, completed Phase 1 study also evaluated the efficacy of EBV-CTLs immunotherapy. However, the response rates for patients with recurrent, metastatic NPC were low: out of 28 enrolled and 21 treated patients, one patient had a complete response with remission for > 8 years (NCT00431210, NCT00834093, Table 1) [84]. The overall data support that EBV-CTLs can have significant impact clinically, but the settings and optimal strategy for expansion and clinical application need to be refined.

Another approach under development by Atara Bio uses allogeneic off-the-shelf T cells expanded from healthy volunteer PBMC. The product, tabelecleucel is in testing in NPC and other settings, where EBV is thought to be a critical driver. In EBV-associated post-transplant lymphoproliferative disease (EBV-PTLD), the T-cell therapy showed remarkable effects, leading to breakthrough approval by the FDA in 2015 and by the European Medicines Agency in 2016 [95]. A single arm study of tabelecleucel plus an anti-PD1 antibody is recruiting (NCT03769467, Table 1) in patients with platinum pre-treated, recurrent/metastatic EBV-associated NPC.

Several other EBV-targeting cell-based immunotherapies are also ongoing in NPC. One clinical trial using a dendritic cell vaccination (CD137L-DC-EBV-VAX) started in 2017 (NCT03282617, Table 1), while three other trials started recruiting in the last two years or will start this year using LMP2 antigen-specific-TCR-T cells (NCT03925896, NCT04509726, Table 1) and EBV-TCR-T cells (YT-E001) (NCT03648697, Table 1). The data for these studies are not yet available. However, the TCR-T cell therapy (NY-ESO-1-specific TCR-T) showed promise in non-HNSCC [96].

An alternative to ex vivo expansion of T cells is vaccination, which aimed to achieve similar results in the patient but without the need for cell manipulation. In 2014, results of a clinical trial using a modified vaccinia ankara (MVA)-based vaccine encoding the EBV antigens EBNA1 C-terminal and LMP2 as a chimeric protein were published. The completed Phase 1 (NCT01147991, Table 1) trial showed safety, with only grade I/II adverse events. An immune response with increased circulating CD4^+^ T cells and antigen-specific T cell responses was reported [82,83]. A separate MVA-based vaccine study targeting EBNA1 and LMP2 led to increased CD4^+^ and CD8^+^ T-cell responses to at least one vaccine antigen in 15 of 18 patients [97]. A Phase 2 study evaluating the safety and efficacy of an adenovirus-LMP1-LMP2 transduced dendritic cell vaccine showed limited efficiency, although the production and administration of the vaccine was successful [98]. Another active, but not recruiting, Phase 2 trial is evaluating the efficacy of an MVA EBNA1/LMP2 vaccine in patients with persistent, recurrent or metastatic nasopharyngeal cancer (NCT01094405, Table 1). Similarly, a recombinant Ad5-EBV-LMP2 vaccine was tested in NPC patients in a Phase 1 clinical trial and was well tolerated (NCT00078494, Table 1). Higher proportions of CD4^+^ T cells were detected in the high dosage group; however, no other functional tests have been done [82].

According to the entries on clinicaltrials.gov, in the last two decades, eight different EBV vaccination studies have been performed, of which five were completed. A recently published review summarises the current state of the whole field of novel immunotherapies in NPC [99]. Overall, T-cell transfer has been the most effective strategy with proof-of efficacy from vaccination outstanding to date.

### 3.3. Human Endogenous Retroviruses, HERV

Recently discovered human endogenous retrovirus (HERV) sequences may offer an additional set of targets for cancer immunotherapy. Those sequences are footprints of human retrovirus exposure in the past. To date, 31 HERV families have been described, and as they are transmitted vertically through generations, approximately 8% of our genome appears to be of HERV origin [100]. HERV has a similar genomic organisation to retroviruses such as HIV and HTLV-1 and have *gag* (group specific antigen), *pol* (DNA polymerase) and *env* (viral envelope) genes. As a result of integration in our genome over tens of millions of years, many of these sequences are altered and have accumulated frameshift mutations and stop codons, which in turn lead to a loss of viral replicative capability [101]. However, some HERV sequences can be expressed in a tissue-specific manner, and the expression is elevated in human cancer. A recent study by the PCAWG consortium found an association of human ERV1 with adverse outcome in renal cancer but did not find such an effect in HNSCC [85]. The HERV-K family of endogenous retroviruses is one of the most studied groups. A high expression of HERV-K-MEL was described in HNSCC, in comparison to healthy tissue [102], and HERV-R (ERV3-1) was reported to lead to radioresistance in HNSCC [103]. Whether the differential expression can be exploited to generate a cancer-type-specific vaccine is currently unknown, but large international efforts, such as that of the PCAWG consortium in the UK from the 100k genomes project [104], will shed light on the breadth of expression and will need to be complemented by studies of T-cell reactivities to HERVs.

## 4. Non-Viral Antigens

### Neoantigens

Mutated antigens (neoantigens) are the by-product of cancer-specific genomic changes resulting in protein changes, which develop and persist during tumour clonal expansion. These neoantigens are not present in non-neoplastic tissues, including the thymus. Therefore, immunological tolerance is less likely. From this backdrop, neoantigens have gained much attention as targets for immunotherapy in recent years. Key hurdles for a mutation to turn into a neoantigen are the transcription, translation into protein, processing of the protein into peptides and then presentation on MHC molecules, which have to be successful. If this does not happen, then T cells are not stimulated to recognise the mutations; however, if even one mutation fulfils these criteria, this could allow immune attack. Potent neoantigens include conventional cancer driver mutations shared among cancer patients, as it is unlikely that the cancer cells can eliminate such mutations in response to immunological selective pressure. Passenger mutations specific to any individual cancer can also be important for the induction of immune recognition by T cells. The Cancer Genomic Atlas (TCGA) and Catalogue of Somatic Mutations in Cancer (COSMIC) are available to researchers to investigate the frequency of cancer mutations in different cancer types. The mutational rate or tumour mutational burden (TMB) in HNSCCs varies, with an average at approximately five mutations/Mb [105,106,107], and no apparent effect of HPV status on TMB [105,106]. However, there does seem to be a significant difference in the types of mutations dependent on HPV status. For example, the most frequently mutated genes in human cancers, tumour-suppressor p53 (TP53) and the proto-oncogene Ras, are more likely to be mutated in HPV^neg^ cases than HPV^pos^ cases [108].

Efforts to exploit such mutations for clinical benefit are ongoing. A Phase 2 peptide vaccine targeting mutant Ras together with IL-2 or GM-CSF in metastatic solid tumours including HNSCC has been completed but no data yet reported (NCT00019331, Table 2). Separately, peptides from mutant TP53 were loaded onto dendritic cells (DCs). This vaccine was evaluated in a Phase 1 trial for HLA-A2/DR4 patients with HNSCC (NCT00404339, Table 2) and was reported to be safe and associated with promising clinical outcome. Immunologically, the authors described decreased regulatory T cells (Treg) levels and vaccine-specific immune responses [109]. A Phase 1 trial (NCT02432963, Table 2) evaluating an MVA vaccine targeting mutated TP53 in combination with pembrolizumab is ongoing to identify its safety and tolerability in patients with refractory TP53 overexpressing cancer including HNSCC.

Not only have conventional shared cancer mutations been targeted by immunotherapy in HNSCCs, but also unique patient-specific mutations have generated increasing interest [110]. Advances in bioinformatics, in the prediction of what features define a ‘good’ MHC I or II binding epitope and better vaccine production technologies have all improved the practicality of targeting neoantigens. Whole exome sequencing (WES) of the cancer tissue and germline (to define the mutations) together with RNA sequencing (to identify which of these variants are then transcribed into RNA) have been applied to identify expressed mutated antigens (mutanome) for individual patients. Several tools including the Immune Epitope Database and Analysis Resources (IEDB, iedb.org) and NetMHC (www.cbs.dtu.dk) are available to predict the binding affinity of epitopes to MHCs. Recent advances in immunopeptidomics can be applied to verify candidate epitopes from tumour cells by confirming presentation in MHC I or MHC II molecules [111]. With the help of such methodologies, researchers can prioritise within a list of potential targets. Four cancer vaccine studies targeting cancer mutanome to treat advanced melanoma and newly diagnosed glioblastoma have been reported recently [72,112,113,114] (NCT02149225, NCT01970358, NCT02035956, NCT02287428). In HNSCCs, Transgene (France) initiated a randomised Phase 1 clinical trial of TG4050, in which an MVA vaccine encoding multiple neoepitopes is made for each patient (NCT04183166, Table 2). The study will evaluate whether vaccination can reduce the chance of recurrence after completed first-line treatment with surgery ± chemoradiotherapy and will also evaluate the durability and breadth of any induced T-cell responses.

Nucleic acid vaccines are also seeing intense evaluation in solid cancers in trials. At the end of 2019, Norwegian company Vaccibody released data from their Phase 1/2 trial DNA vaccine encoding multiple target neoantigens from patients with locally advanced or metastatic cancer, including HNSCCs [81]. Patient treatment started with a single agent anti-PD-1 antibody, while a personalised vaccine was made. Then, the vaccine was tested in patients in conjunction with CD122-agonist (PEGylated interleukin-2, bempegaldesleukin, Nektar Therapeutics, NCT03548467, Table 2). Excitingly, early data suggest that objective responses have been observed by the addition of the vaccine in four HNSCC patients [81]. German company BioNTech, in collaboration with Roche Genentech, are undertaking a very large, multinational personalised cancer vaccine trial in multiple tumour types including head and neck cancer, where the vaccine is given in combination with the anti-PD-L1 antibody azetolizumab (GO39733 trial, NCT03289962). First results of the RO7198457 trial were presented at the virtual ASCO meeting in June 2020 and show the induction of peripheral T-cell responses and a release of pro-inflammatory cytokines in most of the patients. Preliminary data also suggest an infiltration of RO7198457-stimulated T cells in the tumour; however, more evaluation has to be done [115]. The vaccine platform is a nanoparticulate lipoplexed RNA (RNA-LPX) vaccine administered as an intravenous dose and has recently been shown to effectively stimulate immune responses when targeting shared antigens in patients with melanoma [80].

To date, most mutanome-directed studies focussed on targeting single nucleotide variants or insertion/deletions. However, frameshift mutations are now also being exploited, although this work has not yet led to clinical studies. Frame Therapeutics and eTheRNA Immunotherapies have announced a trial where these frameshift mutation sequences are to be delivered using eTheRNA’s TriMix mRNA vaccine platform [116]. Published in 2019, a separate study in a HNSCC patient responding to anti-PD-1 antibody (pembrolizumab) impressively identified T cells specific to a novel in-frame DEK–AFF2 gene fusion [117], suggesting that T cells specific to fusion genes contributed the positive outcome in this patient. This study also further demonstrated the potential to target MYB-NFIB fusion gene, which is a prevalent gene fusion in adenoid cystic carcinomas (ACCs), a rare subtype of adenocarcinoma that commonly arises in the salivary glands [117]. T cells specific to an epitope derived from MYB-NFIB fusion had been identified in one ACC patient with this fusion gene [117].

Collectively, targeting conventional shared cancer mutations to develop off-the-shelf immunotherapy treatment and targeting both shared and specific mutations to develop personalised treatment are promising approaches to treat patients with solid cancers and also have enormous promise in HNSCC.

**Table 2:** Overview of completed and ongoing clinical trials targeting tumour antigens in HNSCC patients.

Drug/Study	Target	Type	Antigen	Additional Drug	Study Phase	Study Start	Patients Estimated/Recruited	HNSCC Patients Enrolled	Study Identifier	Status	Immune Response	Clinical Response	Ref.
Ras Peptide Vaccine	mutant Ras	peptide	shared mutation	aldesleukin, sargramostim	Phase 2	October–97	na	na	NCT00019331	Completed	na	na	
TP53 Peptide Pulsed DC	mutant TP53	dentritic cells	shared mutation	single treatment	Phase 1	September–05	50/17	*n* = 17	NCT00404339	Completed	Decrease of Treg levels; vaccine-specific immunity	safe and promising clinical outcome	[109]
MVA Vaccine Expressing TP53	mutant TP53	MVA	shared mutation	pembrolizumab	Phase 1	November–15	19/12	*n* = 1	NCT02432963	Active, not recruiting	increased p53 spec. T cells	3/11 patients with stable disease	[118]
TG4050	individual mutanome	personalised MVA	neoantigen	single treatment	Phase 1	December–19	30/na	na	NCT04183166	Recruiting	na	na	
VB10.NEO	individual mutanome	personalised DNA	neoantigen	bempegaldesleukin	Phase1/2	April–18	65/na	*n* = 4	NCT03548467	Recruiting	na	4 included HNSCC with stable disease	[81]
RO7198457	individual TAA	RNA	personalised TAA (up to 20)	atezolizumab	Phase 1	December–17	770/572	na	NCT03289962	Recruiting	Release of pro-inflammatory cytokines, peripheral T-cell response	well tolerated	[115]
GL-0810 (HPV16) and GL-0817 (MAGE-A3)	MAGEA3 and HPV16	peptide	Viral Ag/TAA (CTA)	single treatment	Phase 1	na	na/16	*n* = 16	na	Completed	T-cell and antibody responses observed	well tolerated	[66]
Trojan	MAGE-A3 and HPV16-E7	peptide	Viral Ag and TAA (CTA)	single treatment	Phase 1	November–05	90/5	*n* = 5	NCT00257738	Completed	Induction of viral and CTA spec T cells	acceptable toxicity	[67]
Biropepimut-S (GL-0817)	MAGE-A3	peptide	TAA (CTA)	cyclophosphamid	Phase 2	March–17	na/80	na	NCT02873819	Active, not recruiting			
TBI-1201	MAGEA4	TCR T cell	TAA (CTA)	cyclophosphamide, fludarabine	Phase 1	April–14	12/na	na	NCT02096614	Unknown	na	na	
NY-ESO-1 157-165V, 53-62 and 94-102 +CpG 7909	NY-ESO-1	peptide	TAA (CTA)	cyclophosphamid	Phase 1	January–09	42/21	na	NCT00819806	Completed	na	na	
NY-ESO-1b peptide + CpG 7909	NY-ESO-1	peptide	TAA (CTA)	single treatment	Phase 1	September–03	na/9	na	NCT00199836	Completed	na	na	
mixed bacteria vaccine (MBV)	NY-ESO-1	bacterial	TAA (CTA)	single treatment	Phase 1	May–07	12/17	*n* = 1	NCT00623831	Completed	NY-ESO-1 specific antibody and T-cell responses	na	[119]
TBI-1301	NY-ESO-1	TCR T cell	TAA (CTA)	cyclophosphamide, fludarabine	Phase 1	March–15	20/9	na	NCT02366546	Active, not recruiting	na	na	
Anti-NY ESO-1 TCR-Transduced T cells	NY-ESO-1	TCR T cell	TAA (CTA)	cyclophosphamide, fludarabine	Phase 1	April–15	36/na	na	NCT02457650	unknown	na	na	
TAEST16001	NY-ESO-1	TCR T cell	TAA (CTA)	single treatment	Phase 1	April–17	20/6	na	NCT03159585	completed	na	na	
Peptide vaccine	HLA-A24 epitopes derived from LY6K, CDCA1, and IMP3	peptide	TAA (CTA)	single treatment	Phase 2	August–20	40/37	*n* = 37	UMIN000008379	Completed	Antigen-specific CTL responses	Improved DFS and increased OS	[120]
p16 vaccine (P16_37-63)	p16	peptide	TAA	single treatment	Phase 1/2	August–11	na/26	*n* = 26	NCT01462838	Completed	Cellular and humoral immune responses	14/20 tumour response and nine patients with stable disease	[121]
MUC1 peptide plus Hiltonol (Poly ICLC)	MUC1	peptide	TAA	tadalafil	Phase 1/2	April–16	54/16	*n* = 16	NCT02544880	Active, not recruiting	Anti-tumour immune response, decreased macrophages increased TIL	Well tolerated	[122]
TRICOM-CEA(6D) infected DCs	CEA	dentritic cells	TAA	single treatment	Phase 1	January–02	na/14	na	NCT00027534	Completed	na	na	
				single treatment	Phase 1	September–05	na/24	na	NCT00128622	Completed	Depletion of Treg	na	[123]
CEA RNA-pulsed DC cancer vaccine	CEA	dentritic cells	TAA	single treatment	Phase 1	February–97	na/24	na	NCT00004604	Completed	na	na	
INO-1400/1401 DNA vaccine	hTERT	DNA	TAA	INO-9012 (IL12 DNA vaccine)	Phase 1	December–14	54/93	na	NCT02960594	Completed	na	na	
VolATIL	hTERT	peptide	TAA	atezolizumab	Phase 2	February–20	47/na	na	NCT03946358	Recruiting	na	na	
IDO vaccine (IO102)	IDO	peptide	TAA	single treatment	Phase 2	June–20	11/na	all	NCT04445064	Recruiting	na	na	
CAdVEC	Her2	CAR T cell	TAA	single treatment	Phase 1	September–20	39/na	na	NCT03740256	Not yet recruiting	na	na	
CIMAVax Vaccine	EGF	peptide	Cytokine	nivolumab, pembrolizumab	Phase 1/2	December–16	181/na	na	NCT02955290	Recruiting	na	na	

## 5. Tumour-Associated Antigens (TAAs)

### 5.1. Cancer Testis Antigens

The first member of the cancer testis antigen family (CTAs), MAGE-1 (renamed to MAGEA1), was defined by T. Boon’s laboratory in early 1990s as a target for T-cell attack, initially in melanoma but then also in other cancers [124]. Since then, a large body of evidence has accumulated on the expression, biology and immunogenicity of CTAs, of which about 70 gene families and >140 members have been defined [125]. The expression levels of CTAs are of importance in the biological behaviour of many cancers, including progression, metastasis and recurrence [125].

Substantial evidence exists for the expression of CTAs in HNSCCs. MAGE family members are often detected [126,127,128,129,130,131]. In HPV^neg^ HNSCC, the expression of MAGEA1 and MAGEA4 was associated with shorter OS [132,133]. MAGEB6 presented significant association with poor disease outcome, whilst conversely, MAGEA3 and MAGEA6 were reported as a biomarker for longer disease-free survival (DFS) [129]. MAGEA11 mRNA expression was significantly associated with advanced clinical stage and metastasis in lymph nodes [134]. IgG antibody responses against MAGE antigens can be detected in HNSCC patients’ serum [130]. Antibodies to MAGEA1 and MAGED4 were found to be associated with reduced OS in HPV^neg^ HNSCC [132]. It seems most likely that the poor outcome links to the biological function of the CTAs rather than to the immune responses to these molecules. MAGEA3 was targeted in a completed Phase 1 clinical trial to treat patients with advanced HNSCC (NCT00257738, Table 2) using Trojan peptide vaccines composed of CD4 and CD8 epitopes derived from MAGEA3 and HPV16 joined by furin-cleavable linkers and fused to a ‘penetrin’ sequence from HIV TAT [67]. The induction of MAGEA3 and HPV-specific T cell responses detected in TILs and PBMCs demonstrated the immunogenicity of this peptide vaccine [67] with acceptable toxicity but no clinical effect in advanced HNSCC. A randomised Phase 2 study investigating MAGEA3 multipeptide vaccine GL-0817 (Biropepimut-S) in combination with GM-CSF and polyICLC for the prevention of recurrence in HLA-A2 patients with HNSCC is currently ongoing (NCT02873819, Table 2). The vaccine had previously been tested together with an HPV 16 vaccine (GL-0810) (no NCT number). The authors reported specific T-cell and antibody responses in the majority of patients [66]. In an open-label Phase 1 trial, TBI-1201, treatment with T lymphocytes transduced with a MAGEA4-specific TCR gene was tested in HLA-A24 patients with unresectable or refractory MAGEA4 expressing solid tumours including HNSCCs (NCT02096614, Table 2). No result has yet been released.

New York esophageal squamous cell carcinoma 1 antigen (NY-ESO-1) is expressed in numerous cancer types, including HNSCC [126,127,128,129,130,131]. Increased expression of NY-ESO-1 is associated with a higher risk of recurrence, poor response to treatment and shorter survival in HNSCCs [102,133]. Antibodies to NY-ESO-1 can be detected in the sera of ≈6% of HNSCC patients [130]. NY-ESO-1 has been evaluated as an immunogen in many solid tumours clinically, including in randomised trials and using a variety of delivery methods, such as protein + adjuvant, virus-based prime-boost strategies, peptide or nucleic acid vaccines [135]. In a Phase 2 clinical trial of NY-ESO-1 vaccine with ISCOMATRIX adjuvant, strong CD4^+^ and CD8^+^ T cell responses can be detected, indicating that there is a functional repertoire of T cells that can readily be amplified [136]. However, these do not appear to link to clinical responses, and it may be that the induction of Tregs by vaccination may hamper clinical benefit [137]. If this is the case, then conceptually, vaccination against shared antigens will need to take this immunological hurdle into account, for example by systematically removing Tregs in parallel to vaccination. Two completed Phase 1 trials that included HNSCC patients tested synthetic short peptides representing defined NY-ESO-1 HLA-A2 epitopes in combination with CpG 7909 and Montanide ISA-720 (NCT00819806) or Montanide ISA-51 (NCT00199836). CpG 7909 is a well-defined Toll-like receptor (TLR) agonist aimed at innate immune stimulation. The outcome of these two trials has not yet been revealed. A Phase 1 trial using mixed bacteria vaccine (MBV, Coley’s toxins) was undertaken in patients with NY-ESO-1 expressing cancers, including HNSCC (NCT00623831, Table 2); NY-ESO-1-specific antibody and T-cell responses were induced in some vaccinated patients. Inflammatory cytokines such as IL-6, TNF-α, IFN-γ, IL-1β, IL-2 and IL-12 in serum were upregulated in responses to vaccination. Tumour regression was observed in some of the enrolled patients [119]. Since then, investigators also initiated several Phase 1 trials to test NY-ESO-1-specific TCR gene-engineered T cells to treat patients with advanced and metastatic NY-ESO-1 expressing solid tumours. No outcome data are available at the time of writing (NCT02366546, NCT02457650, NCT03159585, Table 2). Lymphocyte antigen 6K (LY6K), cell division cycle-associated protein 1 (CDCA1) and insulin-like growth factor 2 mRNA-binding protein 3 (IMP3) were identified through genome-wide microarray analysis of various cancer tissues and investigated by a Japanese research team [138,139,140]. In a Phase 2 clinical trial (UMIN000008379, Table 2) using multivalent short peptide vaccine targeting HLA-A24 epitopes derived from LY6K, CDCA1 and IMP3, the peptide vaccine significantly improved DFS and increased OS in HLA-A24 patients with advanced refractory HPV^neg^ HNSCC [120]. Synovial Sarcoma/X breakpoint (SSX) family members, SSX1, SSX2, and SSX4 are expressed in the tumour tissue in some patients with HNSCC [130,141]. SSX2-specific antibody responses can found in HNSCC patients’ serum [130], confirming immunogenicity. Other CTA families such as B melanoma antigen (BAGE), G antigen (GAGE), renal tumour antigen (RAGE), sarcoma antigen (SAGE) and X antigen family (XAGE) members are also variably expressed [130,131,142]. No study has yet reported the association of their expression level and clinical outcomes. Cutaneous T-cell lymphoma-associated antigen (CTAGE) members were also reportedly expressed in HNSCCs [143]. There are also many other CTAs that have been reported, including synaptonemal complex protein 1 (SCP1), leucine zipper protein 4 (LUZP4, also known as HOM-TES-85), metalloproteinase inhibitor 3 (MIG-5, also known as TIMP3), disintegrin and metalloproteinase domain-containing protein 17 (ADAM17, also known as TACE), sperm protein associated with the nucleus on the X chromosome (SPANX-CD), cancer/testis antigen 55 (CT55) and IMP1 [129,130,132,144]. The overexpression of ADAM17 and MIG-5 mRNA in HNSCCs was associated with tumour development and progression [144]. Antibody response to IMP-1 was demonstrated as a negative prognostic factor in patients with HPV^pos^ HNSCC [132]. As CTAs are shared non-mutated antigens, central T-cell tolerance may contribute to the inhibition of the generation of autogenous CTA-specific T-cell responses against tumour, and peripheral regulatory cells may limit CTL responses, as observed for NY-ESO-1. The numerous vaccine trials demonstrate the induction of CTA-specific T-cell responses, indicating T-cell tolerance against non-mutant CTAs, can be overcome by a well-designed vaccine. Then, the critical question is whether such T cells, which are detected in the blood, can confer protection in the tumour tissue. A recent study in melanoma suggests that this may be possible: RNA-LPX vaccine encoding MAGEA3, NY-ESO-1, tyrosinase and transmembrane phosphatase with tensin homology (TPTE) were tested in checkpoint-inhibitor (CPI)-treated patients with unresectable melanoma. Antigen-specific polyfunctional T cells were induced in most of the patients, and some of these had a partial responses or stable disease (NCT02410733) [80].

Collectively, in our assessment, CTAs remain useful potential targets for immunotherapies and are worth investigating further. The careful design of studies evaluating the effects not just in the blood but also in the target tissue, i.e., the cancer tissue itself, will be critical to assess what immunological hurdles limit the clinical impact and to pave the way for overcoming these.

### 5.2. Other Tumour-Associated Antigens

The tumour-associated antigens described in this section are non-mutated antigens that are overexpressed in the tumour and, in contrast to the TAA/CTA group are also found in healthy tissue. These are self-antigens, and central tolerance will be generated during T-cell development in the thymus. Central tolerance limits immune response to tumours expressing such TAAs, reflecting the key function of the thymus in preventing autoimmunity. Therefore, if an effective immune response against TAAs is induced, potentially severe immune toxicity may result, and this appears to be borne out by both preclinical and clinical data [145,146].

Some well-studied TAAs, epidermal growth factor receptor (EGFR, also known as ErbB-1 or HER1), carcinoembryonic antigen (CEA), mucin 1 (MUC1), human telomerase reverse transcriptase (hTERT), etc. are also expressed in head and neck cancers at high levels [147,148,149,150,151]. EGFR is one of four members of a family of tyrosine kinase receptors, which also includes ErbB-2,3 and 4 (HER2, 3 and 4). EGFR is activated by binding EGF or transforming growth factor alpha (TGFα); this receptor-ligand binding leads to DNA synthesis, cell proliferation and cancer growth [152]. Thus, the relative overexpression and the growth factor addiction of some cancer types identifies ErbB kinases as appealing treatment targets. Their overexpression in various cancers including HNSCC is often correlated with poor prognosis [148]. Targeting has mainly been through blocking antibodies that stop signalling by inhibiting growth factor binding, which has shown clinical benefit in some patients [153]. Such antibodies can additionally have immunological functions by activating innate immune attack by natural killer cells via antibody-dependent cellular cytotoxicity (ADCC) [154]; alternatively antibody binding can enable complement activation (complement-dependent cytotoxicity, CDC). Treatment with antibodies may further lead to an influx of adaptive immune cells [155], perhaps by the destruction of cancer cells in a way that is immune-permissive. Nonetheless, for the purpose of this review, we will focus on strategies that directly target the growth factor itself or ErbB family members for T-cell attack.

A therapeutic vaccine against EGF was developed in Cuba to reduce the availability of EGF itself (CIMAvax-EGF vaccine) and is now available [156]. This vaccine consists of EGF conjugated with meningitis B bacteria and montanide ISA 51 proteins as adjuvant. It was shown to be safe and may lead to better immune responses and OS in non-small cell lung cancer patients. Intriguingly, toxicities such as skin rashes, electrolyte disorders or eye disturbances, which are common in patients treated with anti-EGFR antibodies, appear to be rare after vaccination (NCT02955290, Table 2).

CEA and its family members are a group of related glycoproteins that play a role in cell–cell adhesion. CEA was first described in human colon cancer in 1965 [157]. Twenty-nine different genes have since been identified in this group, and their amino acid sequences reveal that they belong to the immunoglobulin superfamily [158]. Physiologically, CEA is expressed during foetal development in the gastrointestinal tissue and continues to be expressed in healthy tissues such as the bowel mucosa throughout life. Many epithelial cancers including HNSCC [147] express much higher levels than normal cells, and if that is the case, serum levels have been used to monitor disease progression, e.g., colon cancer. CEA has been investigated as a target for anti-cancer vaccination in many trials. In our own study, we targeted CEA by a DNA fusion vaccine [159]. There was a clear effect of tumour load on immune responses, which is important and relevant here as it is not widely recognised. All patients without measurable disease responded immunologically, while only 60% of patients with measurable disease did so; CEA-specific CD8^+^ T cells were found in 58% and 20% of patients, respectively [159]. Intriguingly, the presence of immune-related organ toxicity, diarrhea after vaccination, identified patients who lived almost three times as long as those without this toxicity. Our data are consistent with the concept that the right vaccine can expand TAA-specific immune responses in spite of central tolerance and that the expression of the target TAAs can cause both toxicity as well as survival benefit [159].

Both protein and RNA-pulsed dendritic cell (DCs) vaccines have completed Phase 1 trials (NCT00027534, NCT00128622, NCT00004604, Table 2). Denileukin diftitox in combination with DCs modified with the fowlpox vector rF-CEA(6D)-TRICOM in patients with CEA-expressing tumours led to the depletion of circulating Tregs (NCT00027534, NCT00128622, Table 2) [123]. A vaccine targeting CEA (Yeast-CEA (GI-6207) increased CD4^+^ and CD8^+^ T cells and downregulated Tregs in some patients (NCT00924092, no HNSCC patients enrolled) [160].

Another example of a shared antigen that is differentially displayed to T cells by cancer cells compared to normal cells is MUC1. Under normal circumstances, MUC1 is a heavily glycosylated protein that forms a protective layer to protect the underlying epithelia from outside influences such as pH differences and microbes [161]. In many malignancies, MUC1 is under- or even de-glycosylated. This leads to an uncovered protein backbone which, after processing and presentation in MHC molecules, enables T-cell attack [162]. Much work has been done preclinically and clinically on evaluating MUC1 in anti-cancer vaccination. In HNSCC, an anti-MUC1/poly-ICLC vaccine was tested with tadalafil in a Phase 1 trial in patients due to undergo salvage surgery (NCT02544880, Table 2) [122]. The trialists had reported previously that tadalafil on its own reduced myeloid-derived suppressor cells (MDSC) and Treg in the HNSCC. This was linked to increase of CD8^+^ TILs, but with no clinical effects. Data from eight patients treated with the combination confirmed these observations, showing an upregulation of PDL1 on non-CD163 expressing immune cells in the tumour, with a loss of CD163^+^PDL1^+^ macrophages at the tumour edges [122] but again with no clinical benefit, leading to the abandoning of a planned Phase 2 study. In contrast, in lung cancer, a randomised study showed that the delivery of MUC1 in an MVA vaccine (TG4010) in the TIME study could lead to clinical benefit [163]. The study is further noteworthy as the authors showed convincingly that vaccination also induced T-cell reactivities against molecules that were not encoded in the vaccine [164]. This concept of ‘epitope spreading’ likely reflects the release of antigen from dying cancer cells, and if it can be reproduced in other trials, it would be an important feature of anti-cancer vaccination, both to be evaluated and as a path to broadening immune attack. The TIME study also suggests that MUC1 is a target worthy of further clinical testing as a vaccine target.

Telomeres are localised at the ends of each chromosome and shorten with each cell replication. This mechanism leads to a protection against cancer in long-lived humans, as after a specific number of cell divisions, the Hayflick limit is reached and a cell becomes postmitotic and undergoes apoptosis. Some cells in the human body can divide an infinite number of times. Examples are embryonic and stem cells. These cells have a high expression of the telomerase reverse transcriptase, which is encoded by the *TERT* (telomerase reverse transcriptase, hTERT in humans) gene. It has been shown that a high expression of hTERT is found in various cancer types. The expression of hTERT gives the cancer cell the ability for limitless replicative potential. Therefore, if hTERT is mandatory for cancer cell survival and expansion, targeting it by immunotherapy is very appealing. This concept appears to be translatable into the clinic. In prostate cancer, a Phase 1/2 study using an hTERT vaccine (UV-1) led to a complete response based on MRI imaging in 45% of the patients with UV-1 specific immune responses [165]. One study investigating an hTERT peptide vaccine (Vx-001) in various cancers, including two HNSCC cases, has shown an induced hTERT-specific immune response [166]. In another vaccination study targeting hTERT using peptide-pulsed DCs, one HNSCC case was included in Phase 2; the authors reported the detection of antigen-specific CTLs assessed by tetramer staining in PBMCs [167]. One Phase 1 clinical trial has been completed in solid tumours including HNSCC, assessing the safety and tolerability of the hTERT DNA vaccine with or without the combination of the IL-12 DNA vaccine (NCT02960594, Table 2). The VolATIL trial is investigating the hTERT vaccine UCPVax and atezolizumab (anti-PD-L1 antibody) in HPV^pos^ HNSCC. For those two studies, no final results are available yet (NCT03946358, Table 2).

The aldehyde dehydrogenase 1 family member A1 (ALDH1A1) was reported as an antigen that can be recognised by CD8^+^ T cells in HNSCC [168]. Additionally, in a separate study, DCs pulsed with ALDH^high^ HNSCC cells lead to sensitised autologous T and B cells, while ALDH^low^ HNSCC cells did not [169]. These data suggest that clinical testing would be warranted.

A further TAA is p16 (*CDKN2A*), which in many cancers, is mutated, deleted, and/or minimally expressed, and therefore leads to uncontrolled cell division and tumour growth [170]. In contrast, in HPV^pos^ cancer, p16 is highly upregulated, as discussed in the HPV section. Using this specific phenotype, one study investigated the effect of vaccination against p16 (p16-derived peptide P16_37-63) in patients with advanced HPV-associated cancers and could show a tumour response in 14 out of 20 patients and stable disease in nine patients; the vaccine was described to induce cellular and humoral immune responses (NCT01462838, Table 2) [121]. A small molecular drug (epacadostat) targets an enzyme called IDO (indoleamine 2,3- dioxygenase). This is an intracellular enzyme in DCs and macrophages and leads to the degradation of tryptophan, which has an effect on T-cell function and survival [171]. Targeting IDO has the aim to regain immune control in cancer tissue. In June 2020, an IDO peptide vaccine started in Phase 2 for HNSCC (NCT04445064, Table 2). This vaccine has been investigated in non-small-cell lung cancer with IDO-specific T cell responses in the peripheral blood, and two of 15 patients were long-term responders [172].

Another interesting preclinical approach is the usage of tumour cell lysates [173]. In this study, DCs pulsed with heat-treated tumour cell lysates of laryngeal cancer could induce anti-tumour immunity against a cell line.

### 5.3. Angiogenesis Targeted by Vaccination

Another attack point for vaccination is angiogenic molecules such as vascular endothelial growth factor (VEGF), PSMA (also known as GCPII, or NAALADase) and tumour endothelial marker 1 (TEM-1, also known as CD248). Cancer cells require continuous angiogenesis to ensure adequate nutrition. The formation of new blood vessels is recognised to be one hallmark of cancer [19], but in contrast to cancer cells, the vascular cells are not genetically abnormal and do not undergo mutations. Therefore, targeting these cells would have the advantage of avoiding immune evasion, which is well recognised in cancer cells [39,40]. It is noteworthy in the context of this review that targeting neo-angiogenesis by immunotherapy, and specifically vaccination, is an attractive treatment option. In HNSCC, a high expression of angiogenesis mediators such as VEGF has been described and is accompanied by worse OS [174,175]. Soluble VEGF can be expressed by cells that experience a lack of oxygen and leads to the dilation of vessels and stimulation of angiogenesis by binding to the VEGF receptor (VEGFR). An anti-VEGF vaccine (CIGB-247) was assessed in advanced solid in a Phase 1 clinical trial with promising results, as it was well tolerated, safe, and was shown to be immunogenic [176], but evaluation in HNSCC has not yet been undertaken.

PSMA is known to be expressed in the prostate endothelium and in prostate cancer and at low levels in healthy tissues [177,178]. However, it is highly upregulated in the neo-vasculature of many of cancers [179,180], and in HNSCC, PSMA staining has been reported to be present in 75% of oral squamous cell carcinoma cases and is linked to a poor survival [181]. While PSMA has been widely tested as a target for imaging in a variety of cancers, most trial data on vaccination comes from patients with prostate cancer, for example using DNA or peptide vaccination [182,183,184]. Curiously, targeting PSMA by vaccination in other cancers and as a strategy to target neoangiogenesis has so far drawn little attention, perhaps reflecting scepticism that anti-cancer vaccination could be useful. Peptide vaccines directed against PSMA and PRAME (preferentially expressed antigen of melanoma) were combined and tested in advanced solid tumours; no HNSCC patient was enrolled. Fifteen of 24 patients showed an immune response assessed by the expansion of PRAME or PSMA-specific T cells. No partial or complete response was seen; however, seven patients showed stable disease for six months or longer (NCT00423254) [185].

A CAR-T cells therapy targeting PSMA is currently under investigation in cervical cancer patients, with no data yet reported (NCT03356795). An anti TEM-1 vaccination was shown to have promising results in preclinical mouse studies, with reduced angiogenesis, increased infiltration of T cells, and tumour control [186].

## 6. Conclusions

Increasing the immunological visibility of cancer cells by training the immune system to recognise tumour-associated or tumour-specific antigens is drawing much interest and many strategies including vaccines and the transfer of T cells expanded in vitro or modified to express chimeric antigen receptors, are being tested. In HNSCC, as in other cancers, the best target(s) remains to be elucidated: the targeting of shared antigens makes production easier, while neoantigens are becoming targetable as new methods for sequencing and vaccine production are making personalised vaccination feasible. Targeting viral antigens, particularly from EBV, is becoming delineated, and definitive trials of efficacy are appearing on the horizon; other antigens, such as HERV and vascular targets are yet to be fully explored. As we are beginning to understand the limitations of checkpoint inhibitor treatments on the one hand, and their modes of action on the other, we predict that future treatment options will combine standard therapies and individualised treatment, and it appears likely that such strategies will also begin to benefit patients with HNSCC.
